# Supplementary figures and images for: Concatenated modular BK channel constructs reveal divergent stoichiometry in gating control by LRRC26 (γ1), pore, and selectivity filter
Source: eLife. 2026 Mar 5;14:RP107681. doi: 10.7554/eLife.107681 (PMC12962649; doi:10.7554/eLife.107681)

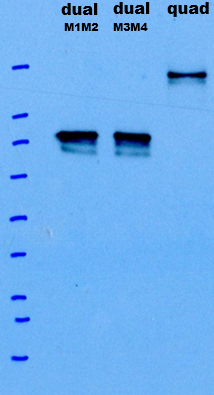

Supplement: Figure 1—source data 1. [file elife-107681-fig1-data1.zip › Figure 1-source data 1.tif]

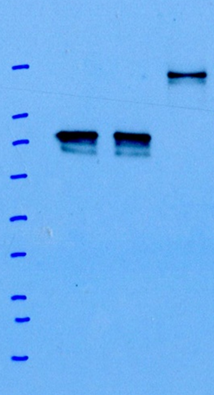

Supplement: Figure 1—source data 2. [file elife-107681-fig1-data2.zip › Figure 1-source data 2.tif]

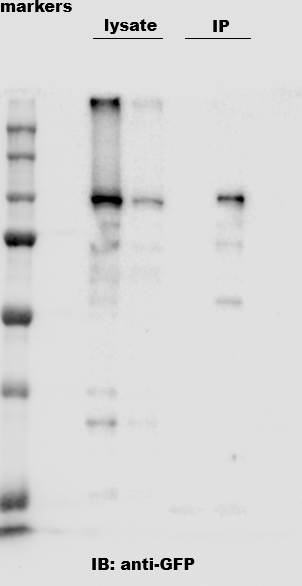

Supplement: Figure 1—figure supplement 1—source data 1. [file elife-107681-fig1-figsupp1-data1.zip › Figure 1-figure supplement 1-source data 1.tif]

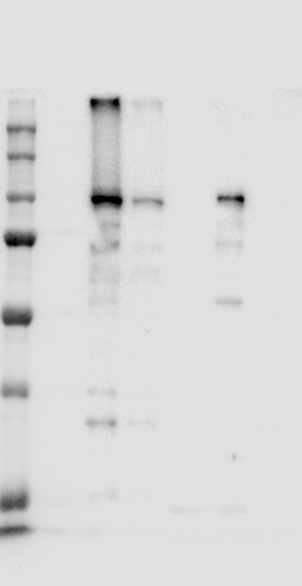

Supplement: Figure 1—figure supplement 1—source data 2. [file elife-107681-fig1-figsupp1-data2.zip › Figure 1-figure supplement 1-source data 2.tif]

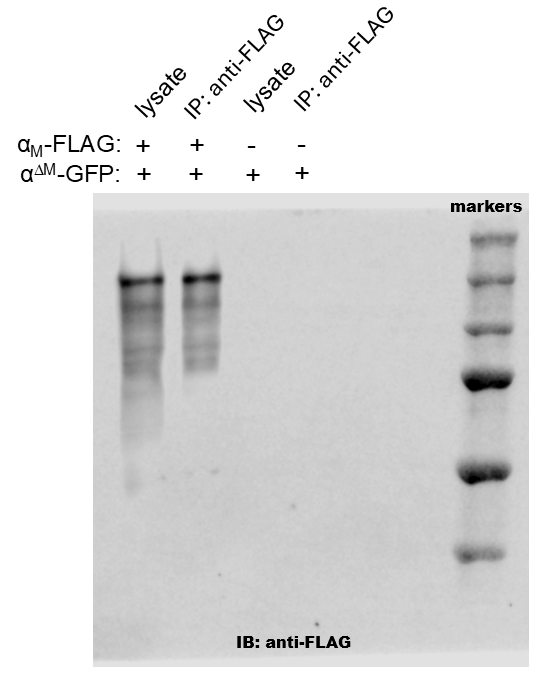

Supplement: Figure 1—figure supplement 1—source data 3. [file elife-107681-fig1-figsupp1-data3.zip › Figure 1-figure supplement 1-source data 3.tif]

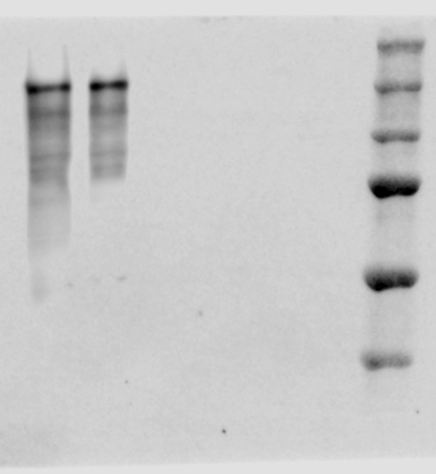

Supplement: Figure 1—figure supplement 1—source data 4. [file elife-107681-fig1-figsupp1-data4.zip › Figure 1-figure supplement 1-source data 4.tif]

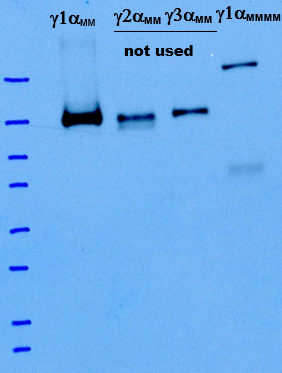

Supplement: Figure 2—source data 1. [file elife-107681-fig2-data1.zip › Figure 2-source data 1.tif]

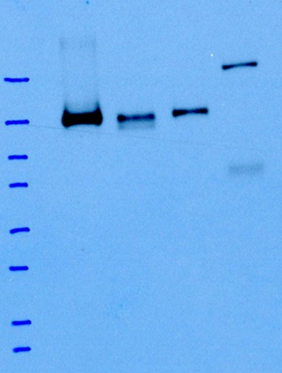

Supplement: Figure 2—source data 2. [file elife-107681-fig2-data2.zip › Figure 2-source data 2.tif]
